# Exoscope-assisted resection of a recurrent left frontal pilocytic astrocytoma

**DOI:** 10.3171/2023.10.FOCVID23158

**Published:** 2024-01-01

**Authors:** Sanjeev Sreenivasan, Danielle Golub, Karen Black, Michael Schulder

**Affiliations:** Departments of 1 Neurosurgery and; 2 Radiology, NorthShore University Hospital and Long Island Jewish Medical Centre, Northwell Health, Manhasset, New York

**Keywords:** exoscope, tumor resection, surgical outcomes

## Abstract

An exoscope strengthens the armamentarium of a neurosurgeon by improving visualization and surgeon ergonomics, reducing surgeon discomfort, and improving coordination among the surgical team. A 23-year-old male patient developed focal seizures and weakness affecting his right arm that was attributable to a recurrent left frontal lesion. Despite two craniotomies at an 8-year interval, chemotherapy, and radiation, the tumor continued to progress. In this video, the authors demonstrate resection of a recurrent left frontal pilocytic astrocytoma with the assistance of an exoscope, neuronavigation, and neuromonitoring. The exoscope can enhance surgical resectability while smoothening the surgical workflow.

The video can be found here: https://stream.cadmore.media/r10.3171/2023.10.FOCVID23158

## Transcript

In this video, we demonstrate exoscope-assisted resection of a recurrent left frontal pilocytic astrocytoma

### 0:28 Case Details.

We present the case of a 23-year-old right-handed male patient who underwent resection of a left frontal lesion in 2014 in another country. This was followed by recurrence after 8 years, for which he underwent craniotomy and resection of lesion at a nearby institution. Initial histopathology was reported as pilocytic astrocytoma with repeat review suggestive of oligodendroglioma without loss of heterozygosity of 1p19q, negative for BRAFv600e.

### 0:55 Past Treatment History.

He underwent a 6-week course of intensity-modulated radiation therapy of 5400 cGy to the lesion. This was accompanied by 4-week adjuvant temozolomide regimen. This period was marked by significant thrombocytopenia, for which chemotherapy had to be discontinued. For radiological persistence of the lesion, he received a tapering course of dexamethasone for the next 4 weeks. Then he noticed worsening right-hand function for the past 1 month. A new MRI showed increase in size of residual lesion with increased cystic change, edema, and calcified component of the lesion.

### 1:28 Radiological Investigations.

The T1 postcontrast images demonstrating superficial component with ring enhancement and nonenhancing core, with deeper core with heterogeneous enhancement along the entire tissue length and breadth. This was suggestive of a superficial cystic or fluid-filled component and deeper solid lesion.

The functional imaging showed poor activation of speech tasks and good activation of finger tapping tasks located on the surface of the lesion. And verb generation tasks were also located close to the surface aspect of the lesion. The perfusion scan showed increased uptake of tracer within the solid core of the lesion. This was suggestive of a recurrent tumor as opposed to pseudoprogression or radionecrosis.

### 2:14 Clinical Decision-Making.

After discussion in neuro-oncology tumor board, the patient was considered a candidate for reresection based on worsening neurological deficit, uncontrolled focal seizures attributable to lesion. The receipt of recent radiation therapy became a contraindication of radiation in this setting.

### 2:32 OR Setup.

The setup of operative room is demonstrated with the surgeon and assistants’ position on the head end of the patient table. The exoscope is positioned behind the surgeon with the arm angled forward. The exoscope video monitor sits at the foot end of the patient. The neuronavigation station also rests at the foot end, and anesthesia table and equipment along the right side of the patient table. This image demonstrates the position of the exoscope behind the surgeon and the arm arching forward with the monitor lying in front of the view for surgeons and assistants.

### 3:01 Surgical Steps.

A cruciate dural incision was made and adhesions of the underlying brain were gently separated from the dura. Neuronavigation was used to confirm position of planned corticectomy, as the lesion was not surfacing.

Neurostimulation was then used to confirm absence of right-sided motor activation within 5- to 10-mm vicinity of planned corticectomy.

A cortical strip electrode was inserted under the dural leaflet, directed posteriorly to map the proximity to the motor cortex. This allowed for ongoing continuous motor evoked potentials during tumor dissection.

A noneloquent region of cortex was identified and, using bipolar forceps, a corticectomy was made.

A cystic structure was gradually encountered in the depth of the corticectomy. The walls of the cavity appeared thick and calcified. A neurostimulation probe was used to identify distance of electrical activity from the walls of the cystic structure. At this point, no motor activity was elicited at 30-mA stimulation.

A friable-appearing area was selected at the floor of the cyst wall and, using bipolar forceps, gradual cauterization with decompression of the lesion was started.

Debulking of the tissue required use of biopsy forceps with coagulation.

Intermittently, a Rhoton dissector was used to separate the calcified tissue plane from the normal-appearing tissue.

The SONOPET ultrasonic suction aspirator was used to debulk the lesion in the depth. This was done after confirming absence of conduction with neurostimulation. As you can see, the tissue was firm and not easily suckable with the SONOPET; hence, debulking required combined use of ultrasonic aspiration and sharp dissection.

Gradual intratumoral decompression was continued using the SONOPET ultrasonic suction aspirator.

Once adequate debulking was achieved, the neurostimulation probe was used to identify proximity to corticospinal tracts. This showed that the posterior margin of lesion was within 5 mm of the tracts and similar proximity was noticed along the anterior, lateral, and medial margins. This was confirmed with neuronavigaton, which showed the position of probe consistent with depth of lesion along all margins.

Hemostasis was secured within the depth of the lesion using cotton balls soaked in peroxide, and Surgicel was used to line the cavity once hemostasis was confirmed.

### 8:01 Postoperative CT Scan.

A postoperative CT scan demonstrated a subtotal resection of the lesion.

### 8:06 Literature on Exoscope-Assisted Neurosurgical Procedures.

Literature has shown that efficacy of surgical resection under the exoscope is similar to that with a microscope.^1^ The exoscope offers a better interaction between the surgeon and assistants and also allows for two surgeons to work in tandem, using four hands, with each surgeon having the same stereoscopic view.^2^

Among series of glioblastomas, it was seen that extent of resection is similar with exoscope and the microscope, and the complication profile also remains the same.^3^ The exoscope is proven to reduce surgeon discomfort while providing excellent delineation of tissue with high resolution.^4,5^

## Disclosures

The authors report no conflict of interest concerning the materials or methods used in this study or the findings specified in this publication.

## Author Contributions

Primary surgeon: Schulder. Assistant surgeon: Sreenivasan, Golub. Editing and drafting the video and abstract: Sreenivasan, Golub, Schulder. Critically revising the work: Sreenivasan, Golub. Reviewed submitted version of the work: all authors. Approved the final version of the work on behalf of all authors: Sreenivasan. Supervision: Schulder. Assisted with imaging: Black.

## Supplemental Information

### Patient Informed Consent

The necessary patient informed consent was obtained in this study.

## References

[1] Di Cristofori A Graziano F Rui CB Exoscopic microsurgery: a change of paradigm in brain tumor surgery? Comparison with standard operative microscope Brain Sci 2023 13 7 1035 37508967 10.3390/brainsci13071035 PMC10377370

[2] Montemurro N Scerrati A Ricciardi L Trevisi G The exoscope in neurosurgery: an overview of the current literature of intraoperative use in brain and spine surgery J Clin Med 2021 11 1 223 35011964 10.3390/jcm11010223 PMC8745525

[3] Baron RB Lakomkin N Schupper AJ Postoperative outcomes following glioblastoma resection using a robot-assisted digital surgical exoscope: a case series J Neurooncol 2020 148 3 519 527 32519286 10.1007/s11060-020-03543-3

[4] Schupper AJ Eskandari R Kosnik-Infinger L A multicenter study investigating the surgeon experience with a robotic-assisted exoscope as part of the neurosurgical armamentarium World Neurosurg 2023 173 e571 e577 36842529 10.1016/j.wneu.2023.02.094 PMC11221417

[5] Fiani B Jarrah R Griepp DW Adukuzhiyil J The role of 3D exoscope systems in neurosurgery: an optical innovation 2021 13 6 e15878 10.7759/cureus.15878 PMC8302823 34327102

